# Hypoxia causes transgenerational impairments in reproduction of fish

**DOI:** 10.1038/ncomms12114

**Published:** 2016-07-04

**Authors:** Simon Yuan Wang, Karen Lau, Keng-Po Lai, Jiang-Wen Zhang, Anna Chung-Kwan Tse, Jing-Woei Li, Yin Tong, Ting-Fung Chan, Chris Kong-Chu Wong, Jill Man-Ying Chiu, Doris Wai-Ting Au, Alice Sze-Tsai Wong, Richard Yuen-Chong Kong, Rudolf Shiu-Sun Wu

**Affiliations:** 1 School of Biological Sciences, The University of Hong Kong, Pokfulam Road, Hong Kong SAR, China; 2 Department of Biology and Chemistry, City University of Hong Kong, Hong Kong SAR, China; 3 Hong Kong Bioinformatics Centre, School of Life Sciences, The Chinese University of Hong Kong, Hong Kong SAR, China; 4 Department of Biology, Hong Kong Baptist University, Hong Kong SAR, China; 5 State Key Laboratory in Marine Pollution, Hong Kong SAR, China; 6 Department of Science and Environmental Studies, The Education University of Hong Kong, Hong Kong SAR, China

## Abstract

Hypoxia is amongst the most widespread and pressing problems in aquatic environments. Here we demonstrate that fish (*Oryzias melastigma*) exposed to hypoxia show reproductive impairments (retarded gonad development, decrease in sperm count and sperm motility) in F1 and F2 generations despite these progenies (and their germ cells) having never been exposed to hypoxia. We further show that the observed transgenerational reproductive impairments are associated with a differential methylation pattern of specific genes in sperm of both F0 and F2 coupled with relevant transcriptomic and proteomic alterations, which may impair spermatogenesis. The discovered transgenerational and epigenetic effects suggest that hypoxia might pose a dramatic and long-lasting threat to the sustainability of fish populations. Because the genes regulating spermatogenesis and epigenetic modifications are highly conserved among vertebrates, these results may also shed light on the potential transgenerational effects of hypoxia on other vertebrates, including humans.

## Abstract (web summary)

Hypoxia has diverse effects on aquatic life. Wang *et al.* show that reproductive defects resulting from hypoxia are epigenetically heritable in Japanese rice fish, and that this intergenerational inheritance is accompanied by differential methylation and gene expression in sperm.

## Graphical abstract

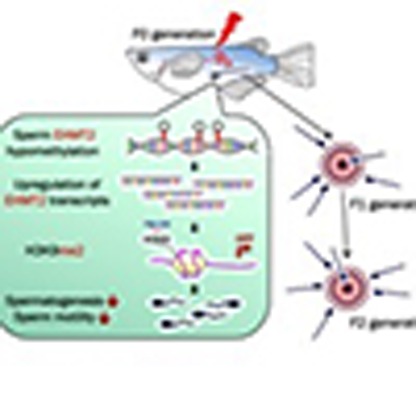

Globally, aquatic hypoxia (a dissolved oxygen (DO) concentration of <2.8 mg l^−1^) is one of the most pressing and widespread problems in both marine^1^ and freshwater^2^,^3^ ecosystems. More than 400 ‘dead zones' have been found worldwide, and climate change will exacerbate the problem in future years. Both laboratory and field studies have demonstrated that hypoxia can affect specific genes along the brain-pituitary-gonad axis, disrupt the synthesis and balance of sex hormones and lead to reproductive impairments and sex alteration in several fish species^4^,^5^,^6^. Notably, hypoxia can also cause similar endocrine disruption and reproductive impairments in rhesus monkeys and humans living at high altitudes^7^,^8^,^9^, and men suffering from hypoxia as a consequence of sleep apnoea exhibit lower levels of male and female sex hormones as well as reduced sex drive^10^,^11^,^12^, showing that hypoxic responses are highly conserved in vertebrates.

Gene expression can be regulated by epigenetic modifications, and epigenetic changes can be passed onto offspring via parental germ-line transmission^13^. Notably, epigenetic changes (for example, DNA methylation, histone modifications and non-coding microRNAs (miRNAs) interference) can also be induced by endocrine disrupting chemicals, thereby leading to transgenerational effects^14^,^15^. For example, F0 mice exposed to vinclozolin during gonadal sex determination display male infertility, and the reduced spermatogenic capacity is inherited by offspring from F1 to F3 (ref. 16) via DNA methylation of the sperm epigenome^17^. Additionally, increases in tumour incidence associated with an altered DNA methylation pattern have been reported in F1 mice after their parents (F0) were exposed to diethylstilbestrol^15^.

Hypoxia is a common phenomenon occurring both in the aquatic environment and in biological systems. It remains unknown if hypoxia causes any transgenerational effects. Arguably, chemicals in F0 can accumulate in germ cells and be passed on to subsequent generations and may partly contribute to the observed transgenerational effects (at least in F1)^18^. However, because hypoxia cannot exist as residues, any transgenerational effects observed could only be attributed to epigenetic changes. If hypoxia induces any transgenerational effects, our current understanding of the environmental and human health risks posed by hypoxia might have been grossly underestimated^19^. Using the marine medaka fish (*Oryzias melastigma)* as a model, the present study tests the hypothesis that hypoxia causes epigenetic changes that perturb the transcriptomic and proteomic profiles of offspring, which ultimately lead to transgenerational reproductive impairments. Jiang *et al.*^20^ demonstrated that the DNA methylome in zebrafish is reorganized in the maternal germ-line but remains stable in the paternal germ-line, which implies that epigenetic alterations in sperm and male progeny are more likely to transmit transgenerational effects. Therefore, this study focuses on epigenetic and transgenerational effects in males.

## Results

### Experimental paradigm

**Figure 1:**
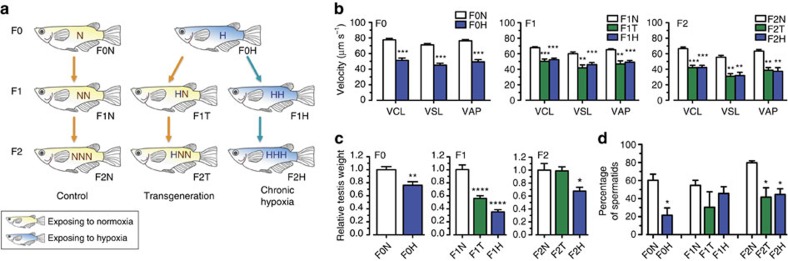
Transgenerational reproductive impairment of the F0, F1 and F2 generations. (**a**) Experimental design. (**b**) Sperm motility in the F0, F1 and F2 generations. N, T and H represent the normoxic, transgenerational and hypoxic groups, respectively. Asterisks denote statistical significance relative to the normoxic group (*n*=8). VAP, average path velocity; VCL, curvilinear velocity; VSL, straight line velocity. (**c**) Effect of parental F0 hypoxic exposure on testis weight and (**d**) percentage of spermatids (*n*=30–55). Data are presented as the means±s.e.m. (*P<0.05, **P<0.01, ***P<0.001 and ****P<0.0001).

Sexually mature F0 marine medaka fish were maintained under normoxia (F0N) or hypoxia (F0H) for 1 month (Fig. 1a). Fertilized eggs from the normoxic controls were continuously raised under normoxia for two generations (F1N and F2N). Fertilized eggs were collected from the hypoxic treatment within 1 h post fertilization (before primordial germ cells had developed^21^) to eliminate any possible effects of hypoxia that are directly exerted on the primordial germ cells. The collected eggs were then divided into two batches: one batch was returned to normoxia and raised for two generations (the transgenerational group, F1T and F2T); and the other batch was continuously raised under hypoxia for two generations (the hypoxic group, F1H and F2H). Histological changes in the testis, sperm motility and fertilization success were measured, and the sperm DNA methylation profile (using methylated DNA immunoprecipitation and high-throughput sequencing, MeDIP-Seq), differential gene expression (using RNA sequencing, RNA-Seq) and proteome profiles (using western blot analysis and iTRAQ quantification) in the testis were determined and compared with the F0–F2 generations in the normoxic, hypoxic and transgenerational groups.

### Hypoxia causes transgenerational reproductive impairments

A significant decrease in the curvilinear velocity (VCL), straight line velocity (VSL) and average path velocity (VAP) of sperm was found in the F0H, F1H and F2H groups, respectively, in which the three generations of fish were maintained throughout the experiment under hypoxic conditions (*P*<0.01). Interestingly, these parameters were also markedly reduced in the F2T transgenerational group despite these offspring (and their germ cells) having never been exposed to hypoxia (Fig. 1b). In parallel, decreases in testis weight (Fig. 1c), percentage of spermatids, and number of spermatids (Fig. 1d) associated with increases in the interstitial space of the testis (Supplementary Fig. 1) were similarly found in the hypoxic groups (F1H to F2H) as well as in the F1T and F2T transgenerational groups. Percentage of fertilization success was also significantly reduced in F1H, F2T and F3H (Supplementary Fig. 2; *P*<0.05). Taken together, our results suggested that hypoxia leads to transgenerational reproductive impairments.

### Hypoxia affects the protein profile

**Figure 2:**
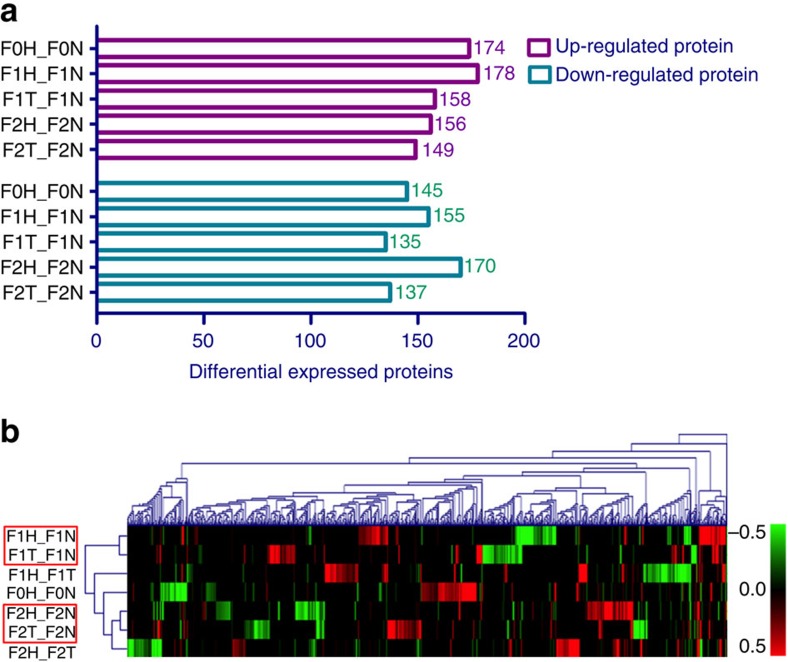
Hypoxia induces differentially expressed proteins (DEPs) in testis of the F0, F1 and F2 generations. (**a**) DEP statistics. *x* axis represents the number of differentially expressed proteins. *y* axis represents the names of comparable groups in different generations. (**b**) Hierarchical clustering heat map (Dendrogram calculated in Euclidean distance and represented by average linkage method). The colour scale represents the fold change in the log_2_ ratio. Red and green bars indicate upregulation and downregulation, respectively. A 1.1-fold change was set as the threshold, and *P*<0.05 was considered to identify significant differences in protein expression (*n*=25–30).

We further studied the proteomes of medaka testis using isobaric tags for relative and absolute quantification (iTRAQ) to relate proteomic changes to the observed phenotypic changes. Proteins identified were aligned to the library of freshwater medaka (*Oryzias latipes*). A total of 5,452 proteins were identified, and significant changes (*P*<0.05) in protein expression among the five groups are shown in Fig. 2a. Hierarchical clustering analysis of the differentially expressed proteins showed that F1T_F1N was in the same cluster as F1H_F1N (marked with a red rectangle) and F2T_F2N was in the same cluster as F2H_F2N (marked with a red rectangle). These results suggest that the observed transgenerational effects were related to hypoxic exposure of F0 parents (Fig. 2b). To better understand the relationship among different treatment groups, the differentially expressed proteins were further classified according to their functions and pathways involved using PANTHER^22^. The analysis showed that most responsive proteins (over 20% of proteins in each group) were nucleic acid-binding proteins primarily involved in the ubiquitin proteasome pathway, which is crucial to the regulation of cellular apoptosis, DNA transcription and extracellular stress responses (Supplementary Data 1). Protein lists were selected based on a cutoff of *P*<0.05 (unadjusted value) for pathway enrichment analyses (Partek Genomics Suite 6.6; Partek, Inc., St Louis, MO, USA). Several altered pathways, including the pentose phosphate pathway, oxidative phosphorylation and glycolysis/gluconeogenesis, were shared between the transgenerational and hypoxic groups across all three generations (Supplementary Data 2). The pentose phosphate pathway is known to play a critical role in defences against oxidative stress in human spermatozoa^23^. The shift of ATP production from oxidative phosphorylation to glycolysis makes energy production less efficient^24^, which may account for the observed reduction in sperm motility.

### Hypoxia induces transgenerational changes in transcriptome

**Figure 3:**
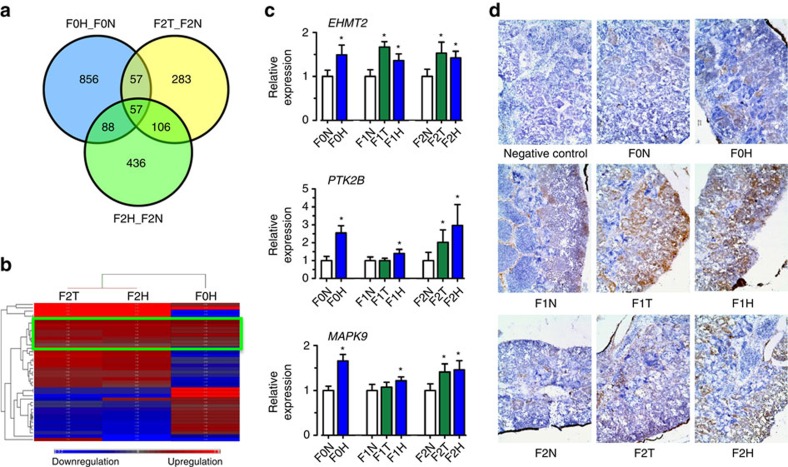
*EHMT2* dimethylated H3K9 found in the transgenerational group by RNA-Seq. (**a**) Venn diagram of the overlapping significant upregulated or downregulated genes shared among F0H_F0N, F2T_F2N and F2H_F2N (*n*=6). (**b**) Clustering of 57 overlapping genes in F0H, F2T and F2H. Red and blue bars represent upregulation and downregulation, respectively (*n*=6). (**c**) Validation of the differential expression of euchromatic histone-lysine N-methyltransferase 2 (*EHMT2)*, protein tyrosine kinase 2B (*PKT2B*) and mitogen-activated protein kinase 9 (*MAPK9*) by qRT-PCR. Data are presented as the means±s.e.m. *P<0.05 (*n*=10). (**d**) H3K9me2 abundance in testicular tissue as revealed by immunostaining. Positive signals are indicated by brownish colouring (*n*=3).

To gain insights into the differential gene expression correlated with impaired sperm function caused by hypoxia, we analysed the transcriptomic changes in testis of the F0 and F2 groups. For the F0 groups, we obtained 37.8 M and 34.5 M quality trimmed reads, which translates to 5.6 Gb and 5.2 Gb of total clean bases for the F0N and F0H groups, respectively. For the F2 group, 20.4 M, 18.8 M and 22.2 M quality trimmed reads yielded 3.1 Gb, 2.8 Gb and 3.3 Gb of total clean bases for the F2N, F2H and F2T groups, respectively. Among the 1,1652 expressed genes, 2,411 were differentially expressed (*P*<0.05) and assigned gene ontology terms. Differentially expressed genes in each group were classified into the categories of cellular component, molecular function and biological process. The distribution pattern was extremely similar among the F0H, F2T and F2H groups (Supplementary Fig. 3). In total, 57 differentially expressed genes overlapped in these three groups, of which 15 genes were co-upregulated and 9 genes were co-downregulated (Fig. 3a; gene list included in Supplementary Data 3). A heat map of the normalized log_2_-fold change was generated for these 57 genes (Fig. 3b). F2T and F2H were highly similar and appeared in the same cluster. The genes commonly upregulated, highlighting the transgenerational effects, are indicated by a green line. Genes consistently upregulated across multiple generations (that is, from F0 to F2), such as protein tyrosine kinase 2B (*PTK2B*), mitogen-activated protein kinase 9 (*MAPK9*) and euchromatic histone-lysine N-methyltransferase 2 (*EHMT2*; Fig. 3c), were identified and further validated by real-time PCR. *PTK2B* is involved in male germ-cell proliferation and maturation^25^. *MAPK9* is a stress-responsive gene that is associated with hypoxic and non-homeostatic conditions^26^. *EHMT2* plays a vital role in DNA methylation and chromatin modification during germ-cell development^27^ and spermatid maturation^28^. *EHMT2* was consistently and significantly upregulated in all three generations in the hypoxic group (F0H, F1H and F2H) as well as in both generations of the transgenerational group (F1T and F2T) compared with the respective generation of the normoxic group (F0N, F1N and F2N; *n*=6–10, *P*<0.05). *EHMT2* upregulation was associated with the elevation in histone H3 lysine 9 dimethylation. H3K9me2 is an important epigenetic marker for gene-silencing induced by hypoxia^29^,^30^. Immunohistochemistry further confirmed an increase in *H3K9me2*-positive cells in both F1 and F2 of the transgenerational and hypoxic groups (Fig. 3d). In addition, Ingenuity Pathway Analysis (IPA) of the 88 deregulated genes in the testes of F0H and F2H in the hypoxic treatment group (Fig. 3a; Supplementary Data 4) highlighted the specific disruption of cell cycle- and apoptosis-related genes through the p53 signalling pathway (Supplementary Data 5). The production of high-quality sperm requires a dynamic balance between cell proliferation, differentiation and apoptosis 31,32. Altogether, these findings suggest that the underlying molecular mechanisms for the observed impairment of sperm motility and quality in the transgenerational (F2T) and hypoxic (F0H, F2H) groups of fish are likely to be different.

### Hypoxia alters the sperm epigenome

**Figure 4:**
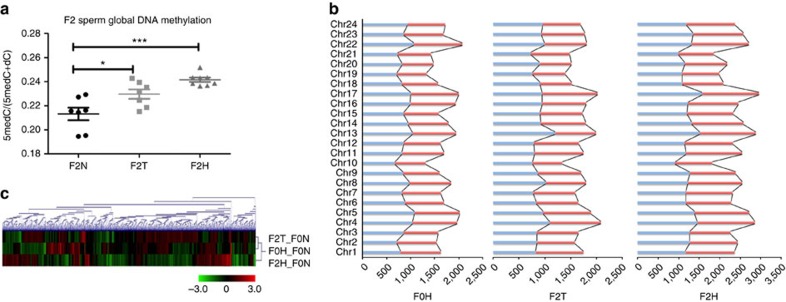
Transgenerational effect of hypoxia on the epigenome. (**a**) The ratio of 5-methylcytosine to the total cytosine pool was measured in the F2N, F2T and F2H groups using liquid chromatography-tandem mass spectrometry (LC-MS/MS; *n*=7–8). (**b**) Clustering of differentially methylated regions (DMRs) in the F0H, F2T and F2H groups compared to the F0N groups. Hypermethylated and hypomethylated regions are represented by red and green colour, respectively (*n*=10). (**c**) Chromosomal distribution of DMRs normalized to the F0N group. Red and blue bars indicate the numbers of hypermethylated regions and hypomethylated regions, respectively. Data are presented as the means±s.e.m. (*P<0.05, ***P<0.001; *n*=10).

Liquid chromatography-tandem mass spectrometry was used to investigate if hypoxia affects global DNA methylation in sperm cells (Fig. 4a). DNA hypermethylation was found in sperm of both the F2T and F2H groups (*n*=7–8), suggesting that sperm epigenetic changes induced by hypoxia can be inherited by fish of the F2T group that have never been exposed to hypoxia. This finding also correlated well with the increase in H3K9me2 in the F2T group described above (Fig. 3d). However, the global DNA methylation changes in F2T and F2H only provided a limited understanding of the molecular mechanisms underpinning the observed transgenerational effects. Hence, methylated DNA immunoprecipitation followed by massively parallel sequencing (MeDIP-seq) was performed to elucidate specific epigenetic alterations in the sperm DNA methylome that can be transferred to the F2 generation. Approximately 50 million paired-end reads containing 3.8 Gb of raw data were obtained for each of the transgenerational, hypoxic and normoxic groups in F0 and F2. The distribution of the differentially methylated regions at the chromosomal level (Fig. 4b) was similar for all three groups (F0H, F2T and F2H), and a similar pattern was also found among the three groups in terms of the types of methylated genomic elements (including intergenic, intron, exon and promoter regions) (Supplementary Fig. 4a). The three groups shared 409 and 2560 genes that were differentially methylated at the promoter and exonic regions, respectively (Supplementary Fig. 4b). Notably, the number of differentially methylated regions was much higher in the F2H group (310 and 879 at the promoter and exon regions, respectively) than in the F2T or F0H groups (154 and 426 for the F2T group at the promoter and exon regions, respectively, and 148 and 432 for the F0H group at the promoter and exon regions, respectively). The methylation changes spanning 24 chromosomes were also larger in the F2T and F2H groups than in the F0H group as indicated by Circos (Supplementary Fig. 4c). The hypoxia-induced changes in the methylation pattern are illustrated in a heat map showing differential methylation at different genomic regions (Fig. 4c). Hierarchical clustering analysis revealed that the methylation pattern of the F2H group was distinct from those of the other groups (F0H and F2T), suggesting that increased exposure to hypoxia was associated with a greater number of methylated regions. The common hypermethylated or hypomethylated regions suggested that these epigenomic changes were hypoxia related and that these changes could be transferred to progeny that have never been exposed to hypoxia.

Through precise epigenome mapping, several genomic regions were found to be differentially methylated in the sperm of both the F0 and F2 generations in the hypoxic and transgenerational groups, which may be responsible for the aberrant sperm motility observed. Specifically, the promoter regions of both *EHMT2* (Supplementary Fig. 5) and *PTK2B* (Supplementary Fig. 6) were hypomethylated, whereas the exonic region of forkheadbox P2 (*FOXP2*; Supplementary Fig. 7) was hypermethylated. The evolutionarily conserved transcription factor *FOXP2* that regulates 300–400 genes in the human genome is involved in germ-cell development and spermatogenesis^33^. *PTK2B* is closely associated with filamentous actin structures and sperm motility, and it plays an important role in fertilization^34^.

### Integrated omics analysis

IPA was carried out to examine the system-wide transgenerational effects of hypoxia in medaka testes by comparing the pool of genes and proteins that exhibited similar patterns of deregulation in F0H, F2H and F2T. Bioinformatic analysis of the deregulated genes (36) and proteins (63; Supplementary Data 6) highlighted the perturbation of several canonical signalling pathways—ATM signalling, Germ-cell-Sertoli cell junction signalling, Sertoli cell–Sertoli cell junction signalling and GADD45 signalling (Supplementary Data 7)—that control testicular function.

**Figure 5:**
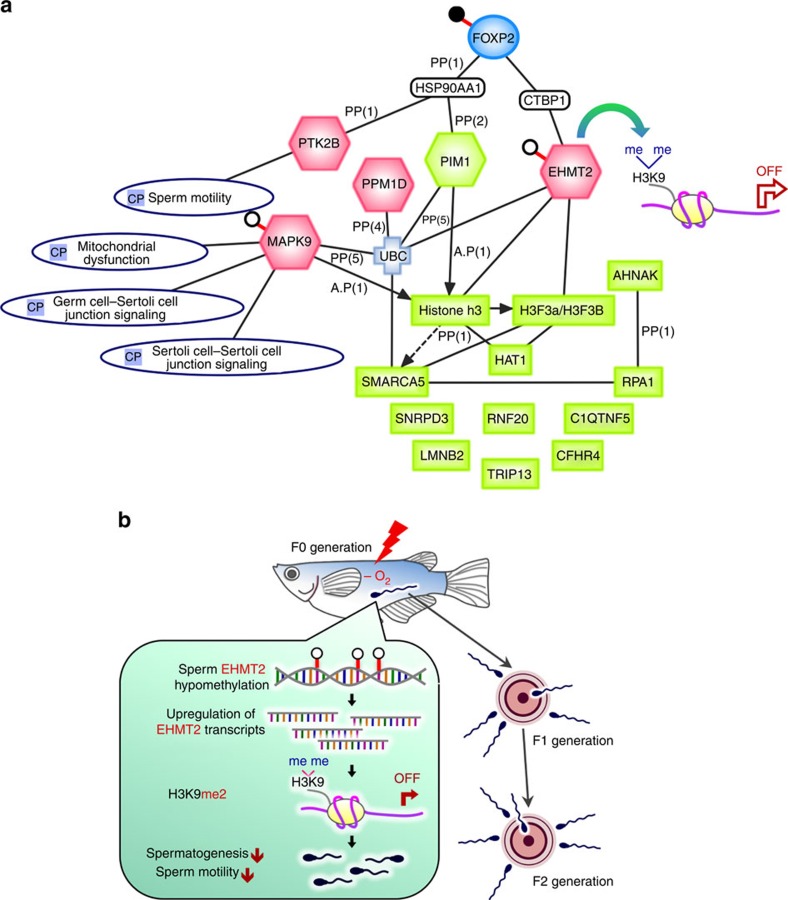
Integrated omics analysis. (**a**) IPA integrates the transgenerational effects at the epigenomic, transcriptomic and proteomic levels, which are represented by oval, hexagonal and rectangular symbols, respectively. Black and white dots indicate hypermethylation and hypomethylation, respectively. Pink and green denote changes in the expression of genes or proteins, respectively. CP, canonical pathways. (**b**) Schematic diagram of the current findings.

Using Circos (Supplementary Fig. 8) and IPA on the integrated ‘omics' data (epigenomic, transcriptomic and proteomic data; Fig. 5a), we further demonstrated that the hypoxia-induced reductions in sperm quality and quantity occurred via *EHMT2*-mediated histone modification. *EHMT2* itself was hypomethylated in the sperm DNA of marine medaka. A number of candidates were highlighted in the pathways analysis. In particular, the *FOXP2* transcription factor was hypermethylated, while *PTK2B*, which is crucial for sperm motility and responds to hypoxic stress and reactive oxygen species, was hypomethylated. These three genes were consistently affected in the F0 through F2 generations, with similar methylation patterns (either hypermethylation or hypomethylation), indicating that the hypoxia-induced epigenetic changes were inherited.

Additionally, we found direct or indirect crosstalk at three levels. Specifically, transcriptome analysis showed repression of the *PIM1* protein kinase and upregulation of the *PPM1D* protein phosphatase under hypoxia. *MAPK9*, predicted to be involved in three canonical pathways related to reproductive and spermatogenic functions (Fig. 5a), was found to be activated^35^, which may explain the reduced sperm motility. However, *EHMT2* is activated through the binding of the oestrogen receptor 1 (*ER1*) and histone deacetylase 1 (*HDAC1*) to myocyte enhancer factor 2A (*MEF2A*) as predicted by IPA. Increased *EHMT2* H3K9 dimethylation (Fig. 3d) was correlated with reduced histone variant H3 family (H3FA/H3FB) and histone acetyltransferase 1 (HAT1) expression. These proteomic analyses collectively indicated that transcriptional silencing signals may occur in the testis of the F2H and F2T groups.

## Discussion

In summary (Fig. 5b), exposure to hypoxia triggers epigenetic changes in the methylome of sperm and alters expression of genes and proteins related to spermatogenesis and gene silencing, leading to a reduction in sperm motility and sperm quantity not only in the F0 generation but also in the F1 and F2 generations that had never been previously exposed to hypoxia (nor their germ cells). Our analyses further suggested that *EHMT2* serves as a regulatory hub and post-translationally modifies histone proteins, such as H3K9 dimethylase, resulting in the downregulation of other chromatin regulators, such as *HAT*, *RPA1* and *SMARCA5*, which may be the underlying mechanisms for the observed transgenerational reduction in sperm motility and number in F1 and F2 (Fig. 5a).

In addition to the genes that initiated transgenerational effects, we also systematically examined hypoxia-responsive genes and proteins in the testes of the F0H and F2H groups by IPA analysis; the results indicated that chronic hypoxia may have disrupted normal sperm production through perturbations of cell cycle control and apoptosis-related processes via the p53 signalling pathway. Apoptosis in the seminiferous epithelium is a key process in sperm production, and a dynamic balance between cell proliferation, differentiation and apoptosis in the testis is essential to ensuring sperm quality^31^,^36^. In particular, apoptosis is important in supporting Sertoli cells, removing defective germ cells and producing high-quality sperm^32^. The observed disruption of testicular apoptosis by hypoxia might reduce both sperm number and sperm quality.

DNA methylation or histone modifications are known to occur in the male germ-line in both fish and mammals, and these epigenetic changes can be passed onto multiple generations of offspring^14^,^37^,^38^,^39^. Whereas sperm cells in mammals undergo reprogramming during spermatogenesis, during which the acquired epigenetic changes may be removed^13^, male medaka fish have a higher chance of inheriting *de novo* epigenetic marks^40^. As such, medaka serves as a good alternative model for epigenetic and transgenerational studies. Moreover, certain epigenetic changes induced by endocrine disrupting chemicals (or hypoxia in this case) are known to be stable and heritable for several generations^41^. Arguably, chemicals can be deposited (and sometimes concentrated) in the germ cells, and their residues can also be passed onto the next generation, which may, in part, contribute to the observed transgenerational effects (at least in F1)^18^. Unlike chemicals, however, hypoxia can only affect the exposed individuals but cannot be carried to the next generation. For the transgenerational group in this study, fertilized eggs produced from F0 were removed from hypoxia before primordial germ cells developed. Thus, all the observed phenotypic changes in F1–F2 of the transgenerational group caused by the exposure of F0 to hypoxia can be solely attributable to transgenerational effects mediated by epigenetic changes. The epigenetic changes and transgenerational reproductive impairments revealed in the present study imply that hypoxia may impose a much more significant and long-term risk to aquatic animals than presently perceived, and potentially to other higher vertebrates, including humans.

In addition to DNA methylation, epigenetic mechanisms such as miRNAs and the post-translational modification of histones may be involved in the observed transgenerational reproductive impairment caused by hypoxia. Although the present report did not provide *in vivo* data to show the involvement of miRNAs, our recent *in vitro* studies demonstrated that miRNAs are involved in the regulation of apoptosis and steroidogenesis in marine medaka gonads in response to hypoxia^42^,^43^. Moreover, our transcriptomic analysis highlighted the deregulation of several genes that code for proteins involved in histone modification, such as Set8a, an important N-lysine methyltransferase that was found to be deregulated in hypoxic medaka testis (Supplementary Data 3)^44^. Further experiments are needed to unravel the underlying mechanisms of how hypoxia can induce DNA methylation in testis, and to understand the possible role of miRNAs and histone modification in the observed transgenerational effect. In addition, further studies may be conducted to test the hypothesis that free radicals^45^ and oxidases (cytochrome *c* oxidase^46^ and monoamine oxidase A (ref. 47)) are involved in the methylation process. In this study, the methylome data were derived entirely from sperm, whereas the transcriptome and proteome profiles were derived from whole-testis samples. As such, the data may not exactly match with each other. In mining the large set of multi-generational data, we focused on the interconnections and the network of genes sharing common biological processes and functions rather than on single genes and proteins. For example, by combining canonical transcriptomic and proteomic pathways according to the activation *Z*-score in IPA, the sperm motility pathway ranks amongst the top 5 (Supplementary Data 8) pathways of all the 545 pathways on the list. Although intergenerational variations were observed, this comprehensive transgenerational study clearly showed that the semen methylome can be affected by hypoxia, which was also supported, at least in part, by the transcriptome and proteome data.

The transgenerational effects observed in this study provide the first evidence that vertebrates can modify their epigenomes in response to hypoxic stress and transfer the epigenetic changes through the male germ-line. These epigenetic changes are associated with altered transcriptomic and proteomic profiles that lead to heritable sperm impairments, which may, in turn, potentially affect the fitness and sustainability of future generations. Thus, the results of this study suggest that hypoxia might pose a dramatic and long-lasting threat to the sustainability of fish populations. Furthermore, because epigenetic changes (especially CG methylation within protein-coding genes) are highly conserved in animals, the result of this study also sheds light on future biomedical research. For example, hypoxic stresses resulting from high altitude and sleep apnoea may also lead to similar epigenetic changes and transgenerational reproductive impairments in humans.

## Methods

### Experimental set-up

All animal research procedures were approved by the Committee on the Use of Live Animals in Teaching and Research (CULATR, #2714-12). All fish used in the experiments were originally obtained from Interocean Industries (Taiwan) and have been maintained in our laboratory for over 10 generations. Two continuous flow systems were set-up inside a controlled environmental chamber (26±1 °C and 14 h light/10 h dark) as previously described in Shang *et al.* 5. In each system, fish were cultured for their entire life cycle (3 months) in five replicate net cages (52 cm length × 18 cm width × 27.5 cm height); each cage contained 45 males and 45 females. Throughout the experiment, the levels of DO for the normoxic and hypoxic systems were maintained at 5.8±0.4 mg l^−1^ and 1.4±0.2 mg l^−1^, respectively. This was achieved by bubbling a constant flow of premixed air and nitrogen mixture into a 300-l reservoir tank through a stripping column (diameter=4 cm) and monitoring DO twice daily using a DO metre (YSI model 580). Other environmental conditions (temperature, salinity, pH and ammonia level) were strictly monitored and controlled to minimize any difference between the treatment groups. The fish were fed to satiation with hormone-free flakes three times per day. Embryos produced by F0 were collected within 1 h post fertilization, before primordial germ-cells developed. The embryos were immediately transferred to normoxic or hypoxic conditions for the development of the F1 generation. The same experimental procedure was applied to the F1 and F2 generations.

### Sample collection

For each generation, adult fish were harvested at 120 days post-hatching, at which time their sex was clearly distinguishable by their external secondary sex characteristics. The histology of F0–F2 testes was also examined (Supplementary Fig. 1). Spermatogonia (∼5–10 μm), primary spermatocytes (∼6 μm), secondary spermatocytes (∼4 μm) and spermatids (∼2 μm) were all found in sexually mature testes in all groups. An equal number of sexually mature male and female fish (*n*=55 each) were randomly sampled and killed after being anaesthetized in ice water. The testis was dissected under a stereo light microscope. The samples were stored at −80 °C for further processing.

### Sperm motility

A total of eight sexually mature males were randomly sampled from each of the normoxic, transgenerational and hypoxic groups at 120 days post-hatching. The testis was removed from each fish and weighed before being gently pressed to extrude semen into 400 μl of artificial seawater for immediate analysis of sperm motility using the CRISMAS image analysis system (Image House, Copenhagen). Swimming patterns were recorded for 10 sperm cells from each fish by capturing 10 different fields for each sperm using a colour CCD camera (Axioplan 2 imaging, ZEISS, Germany). The curvilinear velocity (VCL), straight line velocity (VSL) and angular path velocity (VAP) of fish sperm from the normoxic, hypoxic and transgenerational groups were analysed.

### Quantitative real-time PCR (qRT-PCR)

Differential expression of candidate genes was analysed using qRT-PCR. Total RNA was extracted from the testes of 10 fish using TRIZOL reagent (Invitrogen, Inc., Carlsbad, CA) according to the manufacturer's instructions. RNA concentration was determined using a Nanodrop instrument (Nanodrop 2000). Genomic DNA was removed with an RNase-free DNase kit (Promega, Cat# M6101) by incubating at 37 °C for 30 min. Reverse transcription was conducted using the QuantiTect reverse transcription kit (Qiagen). qRT-PCR of each cDNA was performed using SYBR FAST qPCR Master Mix (Kapa Biosystems, Woburn, MA) on a StepOnePlus Real-Time PCR System (Life Technologies). The PCR assay contained 1:25 diluted reverse transcription (RT) products, 1 × Master Mix and 200 nM of each primer, and it was performed with a 3-min initial denaturation at 95 °C followed by 40 cycles of 95 °C for 5 s and 60 °C for 20 s. The 18S ribosomal RNA housekeeping gene was used for internal normalization for each gene. To calculate gene expression, the comparative Ct method (ΔΔCt) was used. One-way analysis of variance (ANOVA) was used to test for differences between groups. Statistical significance was set at *P*<0.05. Each group included 10 replicates (*n*=10). The primer sequences of the selected genes were designed using the online Primer 3 programme (http://frodo.wi.mit.edu/). The following gene-specific primers were used: *18S*-forward, 5′-gacaaatcgctccaccaact-3′; *18S*-reverse, 5′-cctgcggcttaatttgaccc-3′; *EHMT2*-forward, 5′-caccaa acagcacgagacaa-3′; *EHMT2*-reverse, 5′-ctggttgttgttggccatga-3′; *MAPK9*-forward, 5′-ttccagggcacagatcacat-3′; *MAPK9*-reverse, 5′-gttcctcaccgtctccatca-3′; *PTK2B*-forward, 5′-gtccaaagcagagca acaca-3′; and *PTK2B*-reverse, 5′-cttccccaggttgttgttgg-3′.

### Quantification of genomic DNA methylation in sperm

Each testis was gently squeezed, and the released semen was collected. Sperm was obtained by centrifugation (1,500 r.p.m.), and for each treatment, sperm DNA was extracted from 7 to 8 fish following the protocol of the CpG Methylquest DNA Isolation Kit. Briefly, 1 μg of DNA was measured via Nanodrop (Nanodrop 2000) and digested using DNA Degradase Plus (Zymo research) for 1 h at 37 °C. The digested samples were then dissolved in buffer containing 5% of 0.1% formic acid in methanol and 95% of 0.1% formic acid in deionized water. A 10-μl aliquot of each DNA digest sample was separated on a Hypersil Gold C18 Column (2.1 mm × 20 mm, 1.9 μm, Thermo) using the HP1100 series HPLC system (Agilent Technologies). The mobile phase consisted of 0.1% (v/v) formic acid/water and 0.1% (v/v) formic acid/methanol with a linear gradient increase of 1.5%/min of the methanol phase from 0 to 22.5% at 15 min. Liquid chromatography separation was performed at a flow rate of 0.2 ml min^−1^, and the elution was analysed using a AP3200-QTRAP mass spectrometer (AB SCIEX). The collision energy was set at 14 V for all the analytes. Quantification was conducted in multiple reaction monitoring mode for the transition pair of *m*/*z* with (molecular ion)/(fragment ion) of 242.1/126.3 for 5-methyl-2′-deoxycytidine (5 mdC) and 268.1/152.3 for deoxycytidine (dG) [deoxyguanosine (dG)=5 mdC+2′-deoxycytidine (dC)]. The recorded spectra were analysed using Analyst 1.5.2 software (AB SCIEX). Data are presented as the means±s.e.m. *P<0.05, ***P<0.001 and *n*=7–8 for each group.

### Methylated DNA immunoprecipitation-sequencing of sperm

For each group, sperm from 10 male fish were collected by gently pressing the harvested testis and pooled. Sperm genomic DNA was extracted using a DNA extraction kit (MO BIO Ultraclean Tissue & Cells DNA Isolation Kit, USA). Extracted DNA was prepared by the Paired-End DNA Sample Prep Kit (Illumina) according to the manufacturer's instructions. Briefly, DNA was sonicated to fragments of 100–500 bp before the addition of dAs and adaptor ligation. Methylated DNA was then pulled down by an antibody specifically recognizing 5-methylcytosine. The pull-down fraction containing the antibody-5-methylcytosine complex was subjected to column purification (Zymo Research). The purified DNA was next enriched by an adaptor-mediated PCR process. Fragments of 220–232 bp were gel purified with a Gel Extraction Kit (Qiagen). An Agilent 2000 analyser was used to quantify the DNA before proceeding to library preparation and Illumina Hiseq 2000 sequencing at the Beijing Genomics Institute (Wuhan, China). Qualified raw data were provided by the Beijing Genomics Institute in FASTQ format. The genome sequence of freshwater medaka (*Oryzias_latipes* release 73 downloaded from ensemble) was used as the reference genome for bowtie alignment. DiffReps software^48^ was employed to identify the differentially methylated regions with a false discovery rate (FDR) cutoff of 0.01.

### Whole-testis transcriptome sequencing

Total RNA was isolated from six testes of fish from each treatment group using TRIZOL reagent (Life Technologies, CA, USA). Three testes were pooled as one biological sample, and these two pooled replicates were used for library construction. RNA concentrations were measured using a Qubit RNA Assay Kit on a Qubit 2.0 Fluorometer (Life Technologies, CA, USA). A quantity of 300 ng of total RNA with an RNA Integrity Number >8, as determined by the Agilent 2100 Bioanalyzer system, was used for library construction (Agilent Technologies, CA, USA). Ten independent libraries were prepared for RNA sequencing. Briefly, the cDNA libraries were prepared using the TruSeq Stranded mRNA LT Sample Prep Kit (Illumina, San Diego, USA) following the manufacturer's recommendations. Index codes were ligated to identify the individual samples. mRNA was purified from total RNA using poly-T oligo-attached magnetic beads (Illumina, San Diego, USA) before fragmentation using divalent cations under elevated temperature in Illumina fragmentation buffer. First- and second-strand cDNAs were synthesized using random oligonucleotides and SuperScript II followed by DNA polymerase I and RNase H treatment. Overhangs were blunted by treatment with exonuclease/polymerase followed by 3′-end adenylation and ligation to Illumina PE adaptor oligonucleotides. DNA fragments successfully ligated with adaptor molecules on both ends were enriched using the Illumina PCR Primer Cocktail in a 15 cycle PCR reaction. Products were purified using the AMPure XP system and quantified using the Agilent Bioanalyzer 2100 system. Before being subjected to sequencing, the libraries were normalized and pooled together in a single lane on an Illumina MiSeq platform. Paired-end reads of 150 bp were sequenced. Sequence reads were trimmed dynamically according to BWA's −q algorithm. Briefly, a running sum algorithm was executed that generated cumulative area plots from the 3′- to the 5′-end of the sequence reads where positions of base-calling Phred quality lower than 30 caused an increase in area and vice versa. Plots were constructed for each read individually. The read was trimmed from the 3′-end to the position where the area was greatest. Read pairs were then synchronized such that all read pairs with sequences on both sides longer than 35 bp after quality trimming were retained and any singleton reads resulting from read trimming were removed. All the downstream analyses were based on quality trimmed reads^49^.

Sequencing reads were mapped to the *Oryzias melastigma* testis-specific transcript assembly as described previously^50^ using Novoalign v3.00.05 (http://www.novocraft.com/) with parameter –r ALL to report all multi-mapped reads. Alignment files were parsed using Samtools. Briefly, the number of reads mapping to each transcript in each sample was summarized to generate a count table. Read count data were then subjected to differential expression analysis using the EdgeR R package^51^. Samples of identical treatments were considered as biological replicates. B&H corrected *P* value<0.05 and fold change>1.2 were set as the threshold for significant differential expression.

### Whole-testis proteomics quantification

Testes from 25 to 30 fish from each group were pooled and homogenized in lysis buffer (2 M thiourea; 4% CHAPS; 7 M urea; and 40 mM Tris-HCl, pH 8.5) and reduced with 10 mM DTT for 1 h at 56 °C. Samples were alkylated with iodoacetamide (55 mM final concentration) in the dark for 1 h. The treated samples were precipitated in cold acetone overnight at −20 °C. After centrifugation at 30,000 rcf, the pellet was dissolved in 0.5 M triethylammoniumbicarbonate (TEAB; Applied Biosystems). After Bradford assay for protein quantification, protein (30 μg) was separated by 12% SDS–polyacrylamide gel electrophoresis and then digested with trypsin gold. After digestion, the samples were labelled with the iTRAQ Reagent 8-plex Kit according to the manufacturer's protocol. The labelled peptides were fractionated by strong cation exchange chromatography, and the eluted fractions were analysed by LCMS-MS Triple TOF 5600 (AB SCIEX). These steps followed a previously described method^52^. The acquired raw data (.wiff) were converted to a different format (.mgf) using AB SCIEX MS Data Converter software. The MS/MS spectra were queried against the protein database of freshwater medaka (*Oryzias latipes*) with MASCOT software (Matrix Science, London, U.K). Software Scaffold (Proteome Software Inc., Portland, OR, USA) was used to quantify and analyse the data derived from the MS/MS intensities of the reporter tags. A 1.1-fold change was set as the threshold, and *P*<0.05 was used to identify significantly expressed proteins. Gene ontology classification was studied using PANTHER (http://pantherdb.org/), and pathway enrichment was conducted by Partek Genomics Suite 6.6 (Partek, Inc., St Louis, MO, USA).

### Histology

The testes of three fish from each treatment group were sectioned for immunohistochemistry studies and histological examination after haematoxylin and eosin staining. Immunohistochemistry was performed on 8-μm paraffin-embedded fish tissue sections (H3K9me2–1:500; Cell Signaling, USA). Slides were processed with the Bondmax Immunohistochemistry Staining System (Leica, Bannockburn, IL, USA) according to the recommended procedure. Finally, the slides were dehydrated in pure ethanol and covered in mounting medium for imaging (Nikon 80i). The slides were also stained with haematoxylin and eosin to study testis morphology according to a previously described protocol^53^.

### Integrated analysis and statistics

IPA was used to combine proteomics and transcriptomics results according to the IPA database under the ‘core analysis' section for each data set and the ‘comparison' section for integration. The *Z*-score was calculated according to the biological process involved. Positive and negative Z-scores represented increasing or decreasing biological processes, respectively. Integrative Multi-species Prediction (http://imp.princeton.edu/) was used to predict protein–protein interactions and to link shared pathways among proteins of interest.

The Circos figure was based on the comparison among F2H_F2N, F2T_F2N and F0H_F0N, including MeDIP-seq, transcriptomics and proteomics data. For MeDIP-seq data, the histogram represents the log2 (fold change) of the methylation state at different positions of the 24 chromosomes. Yellow and blue bars represent hypermethylation and hypomethylation, respectively. For transcriptomics data, the histogram represents the log2 (fold change) of transcript level for significant genes (*P*<0.05). Red and green bars represent upregulation and downregulation, respectively. For proteomics data, the scatter plot represents the log2 (fold change) of protein expressions. Red and green points represent upregulation and downregulation, respectively. Protein names are labelled in green or red text in the figure. Shared protein between protein and RNA-Seq data are highlighted in a larger font size. The figure was generated using Circos version 0.67, and the genomic coordinates of the genes were identified using the *HdrR* genome (Oct 2005; retrieved from Ensembl).

Statistical analyses of gene expression profiles were performed using GraphPad Prism 6 (GraphPad Software, San Diego, CA). One-way ANOVA was used to test for differences among groups. Normality and homogeneity of variance (F-test) were assessed (*P*≥0.05) before analysis. If *P*<0.05 according to the F-test, a Kruskal–Wallis test was then used. A Dunnett test was further applied to identify significant differences between groups if *P*<0.05 according to the ANOVA or the Kruskal–Wallis test.

### Data availability

The RNA sequence data from this study have been submitted to the NCBI Sequence Read Archive (SRA) (http://www.ncbi.nlm.nih.gov/sra) under the accession number SRP074531. The MeDIP sequencing data are available in GEO under accession code GSE81312.

## 

## Additional information

**How to cite this article:** Wang, S.Y. *et al.* Hypoxia causes transgenerational impairments in reproduction of fish. *Nat. Commun.* 7:12114 doi: 10.1038/ncomms12114 (2016).

## Supplementary Material

Supplementary Information Supplementary Figures 1-8

Supplementary Data 1 This file shows the classification of differentially expressed proteins according to their functions and pathways involved using PANTHER analysis.

Supplementary Data 2 This file demonstrates the significant altered pathways in F0, F1 and F2 generations.

Supplementary Data 3 This file shows the differentially expressed genes and overlapped DEGs in F0H, F2T and F2H.

Supplementary Data 4 This file shows the list of the 88 common deregulated genes in testes of the F0H and F2H groups.

Supplementary Data 5 This file shows the result of IPA analysis which is used to determine the hypoxic effects on testes.

Supplementary Data 6 This file shows the list of genes (36) and proteins (63) which exhibits similar patterns of deregulation in F0H, F2H and F2T.

Supplementary Data 7 This file demonstrates the result of Ingenuity Pathway Analysis by using the common deregulated genes and proteins in F0H, F2H and F2T.

Supplementary Data 8 This file shows the Ingenuity Pathway Analysis by combining canonical transcriptomic and proteomic pathways according to the activation z-score.
