# Supplementary material for: Hypoxia causes transgenerational impairments in reproduction of fish
Source: Nat Commun. 2016 Jul 4;7:12114. doi: 10.1038/ncomms12114 (PMC4932196; doi:10.1038/ncomms12114)
Supplement: Supplementary Information — Supplementary Figures 1-8 [file ncomms12114-s1.pdf]

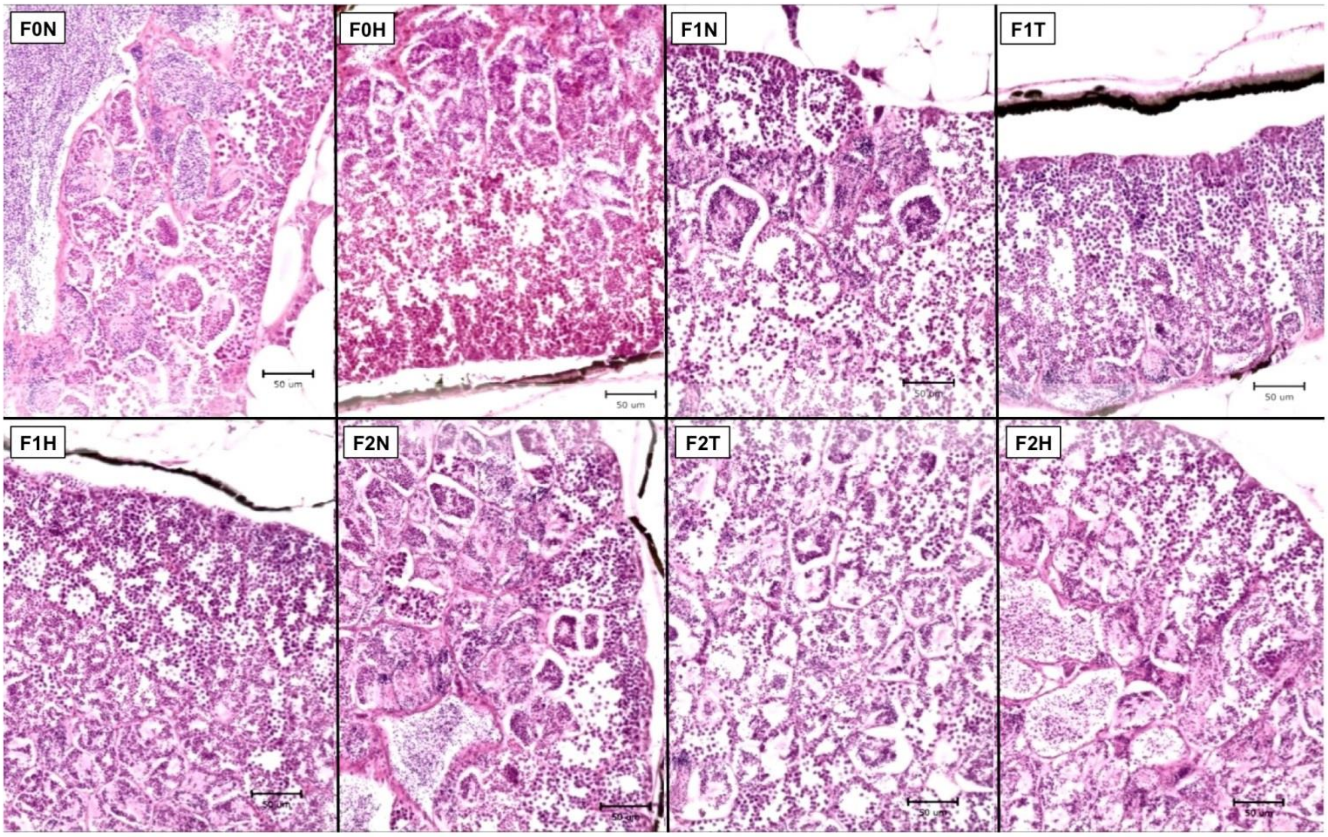


**Supplementary Fig. 1. Effect of parental F0 hypoxic exposure for one month and the corresponding testicular histological examination in the F0, F1 and F2 generations.** The figures show seminiferous tubules containing germ cells at different stages of differentiation (n=3). Bar = 50 µm.

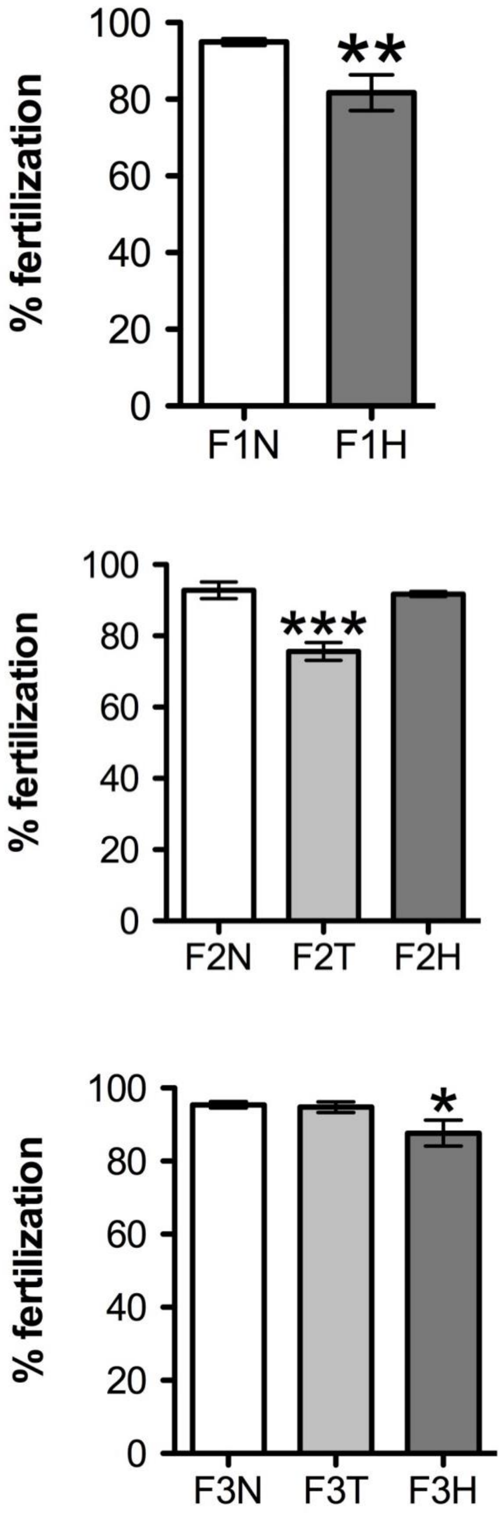


**Supplementary Fig. 2. Effect of hypoxic exposure on % fertilization in F1 (a), F2 (b) and (c) F3 embryos in the normoxic controls vs transgenerational and hypoxic treatment groups.** Data are presented as the means ± S.E.M. Asterisks denote statistically significant differences compared to the normoxic control (*$p < 0.05$, ***$p < 0.001$, $n = 3 – 6$).

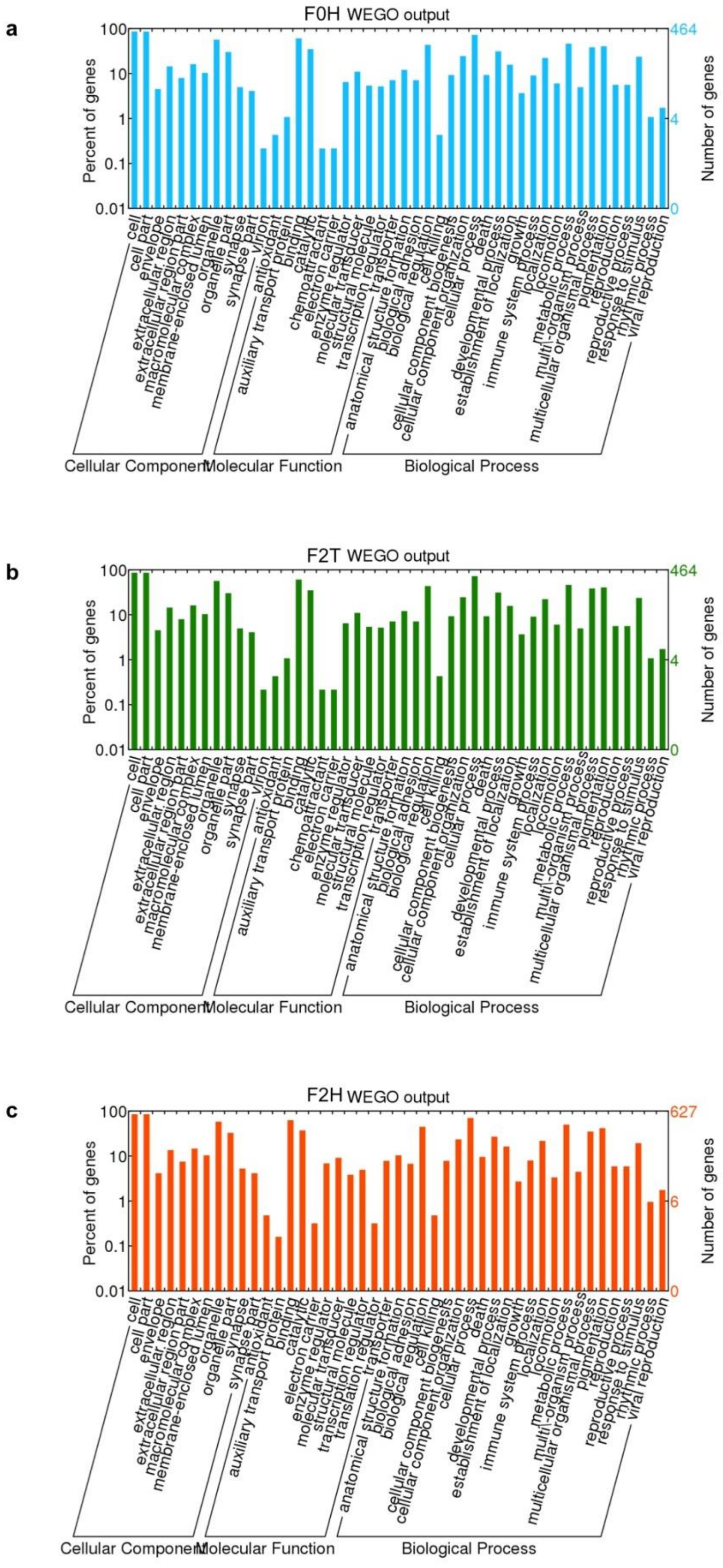


**Supplementary Fig. 3. Gene ontology analysis of differentially expressed genes in the (a) F0H, (b) F2T and (c) F2H generations.** Differentially expressed genes in each group were classified into the categories of cellular component, molecular function and biological process.

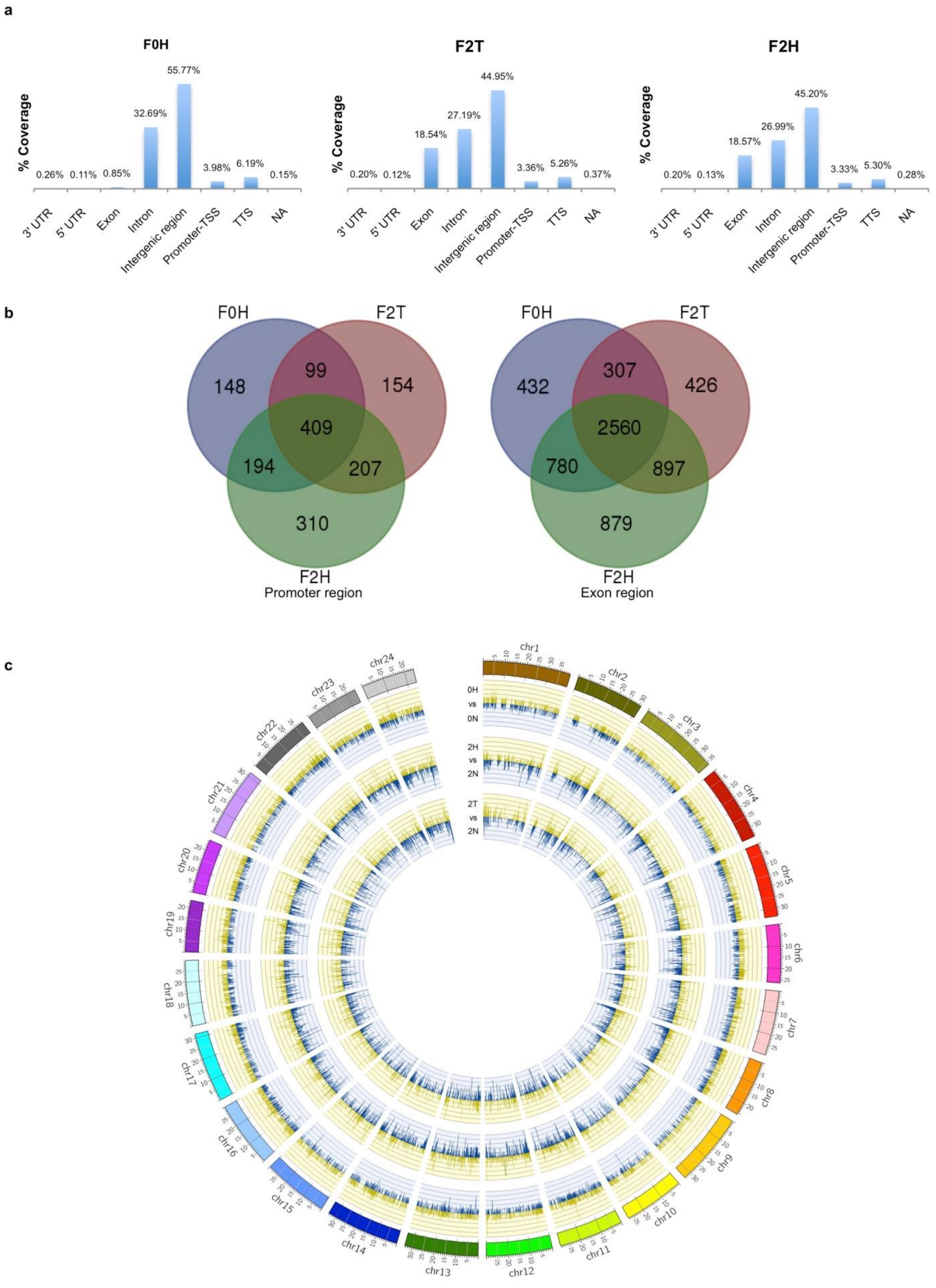


**Supplementary Fig. 4. Effect of hypoxia on the methylation profile.** (a) Bar charts representing the proportions of hypermethylated or hypomethylated CpGs in regions containing different genomic features. Promoter-TSS is defined as regions within ± 1,000 bp of transcriptional start sites. (b) A Venn diagram displaying the number of differentially methylated genes in promoter and exon regions. (c) Circos diagram showing differentially methylated regions in 24 chromosomes of marine medaka. Yellow and blue bars represent hypermethylated and hypomethylated regions, respectively (n = 10).

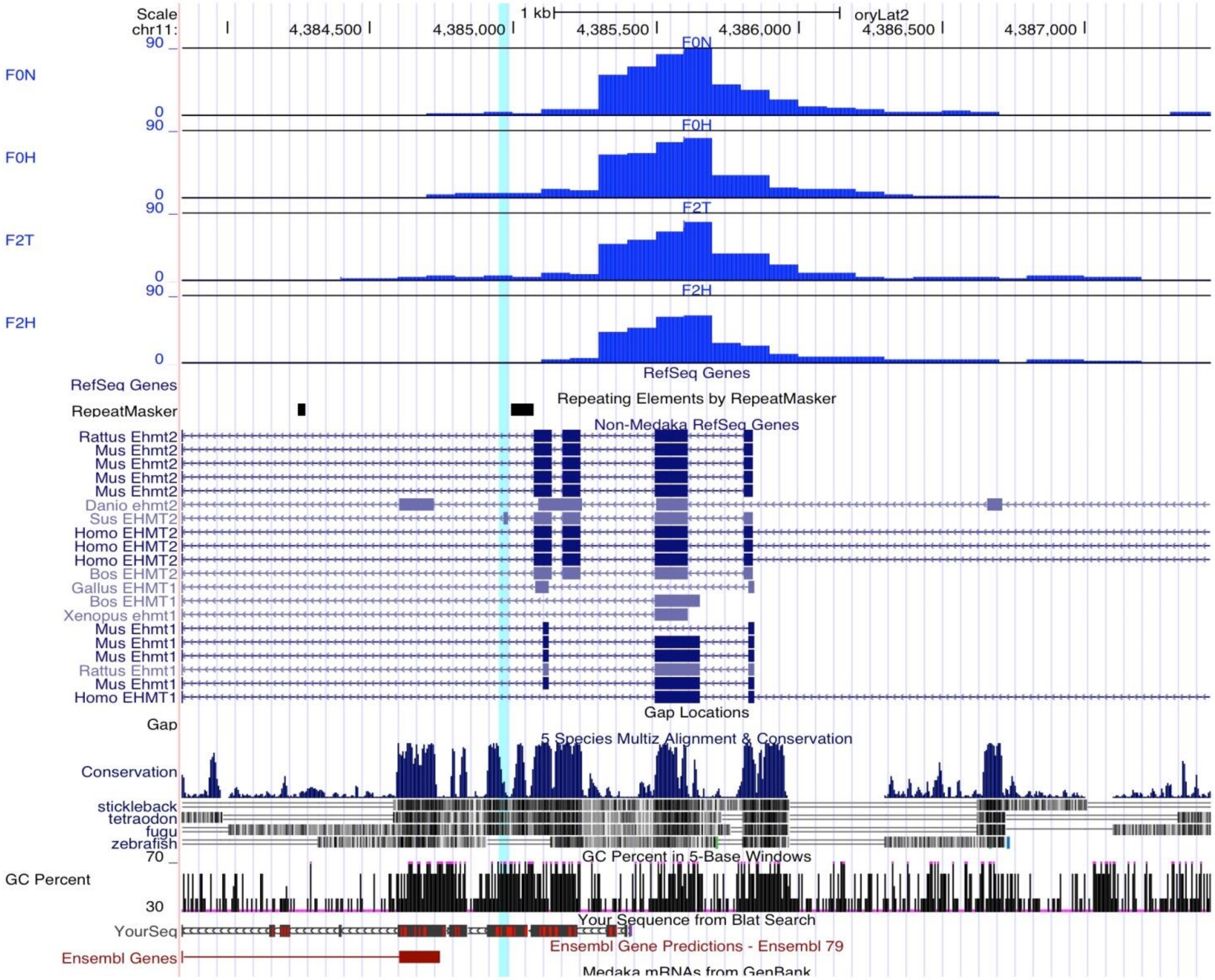


**Supplementary Fig. 5. Differentially methylated regions of EHMT2 in the sperm of generations F0 and F2.** A snapshot of MeDIP-seq results obtained using the UCSC genome browser for the genome-wide analysis. The box plot represents DNA methylation signals along the chromosome. Cut-off criteria were calculated using EdgeR. EHMT2 displayed a 1,513-bp region in the promoters and the first exons of F0H, F2T and F2H that was hypomethylated compared to the normoxic control groups ($n = 10$; $p < 0.01$).

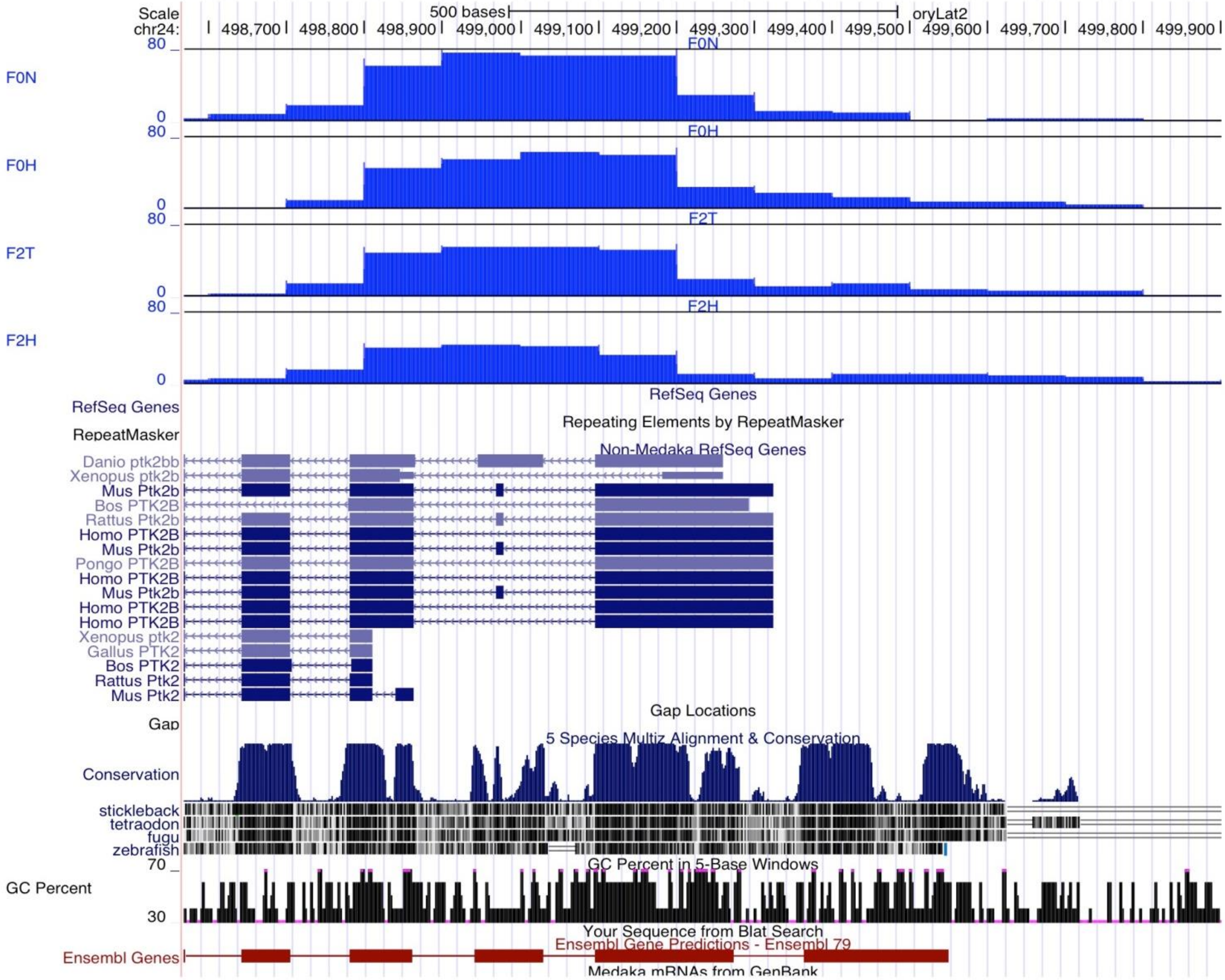


**Supplementary Fig. 6. Differentially methylated regions of PTK2B in the sperm of generations F0 and F2.** A snapshot of MeDIP-seq results obtained using the UCSC genome browser for the genome-wide analysis. The box plot represents DNA methylation signals along the chromosome. Cut-off criteria were calculated using EdgeR. PTK2B displayed a 1,203-bp hypomethylated region in the promoters and the first exons of F0H, F2T and F2H that was hypomethylated compared to the F0N normoxic control groups ($n = 10$; $p < 0.01$).

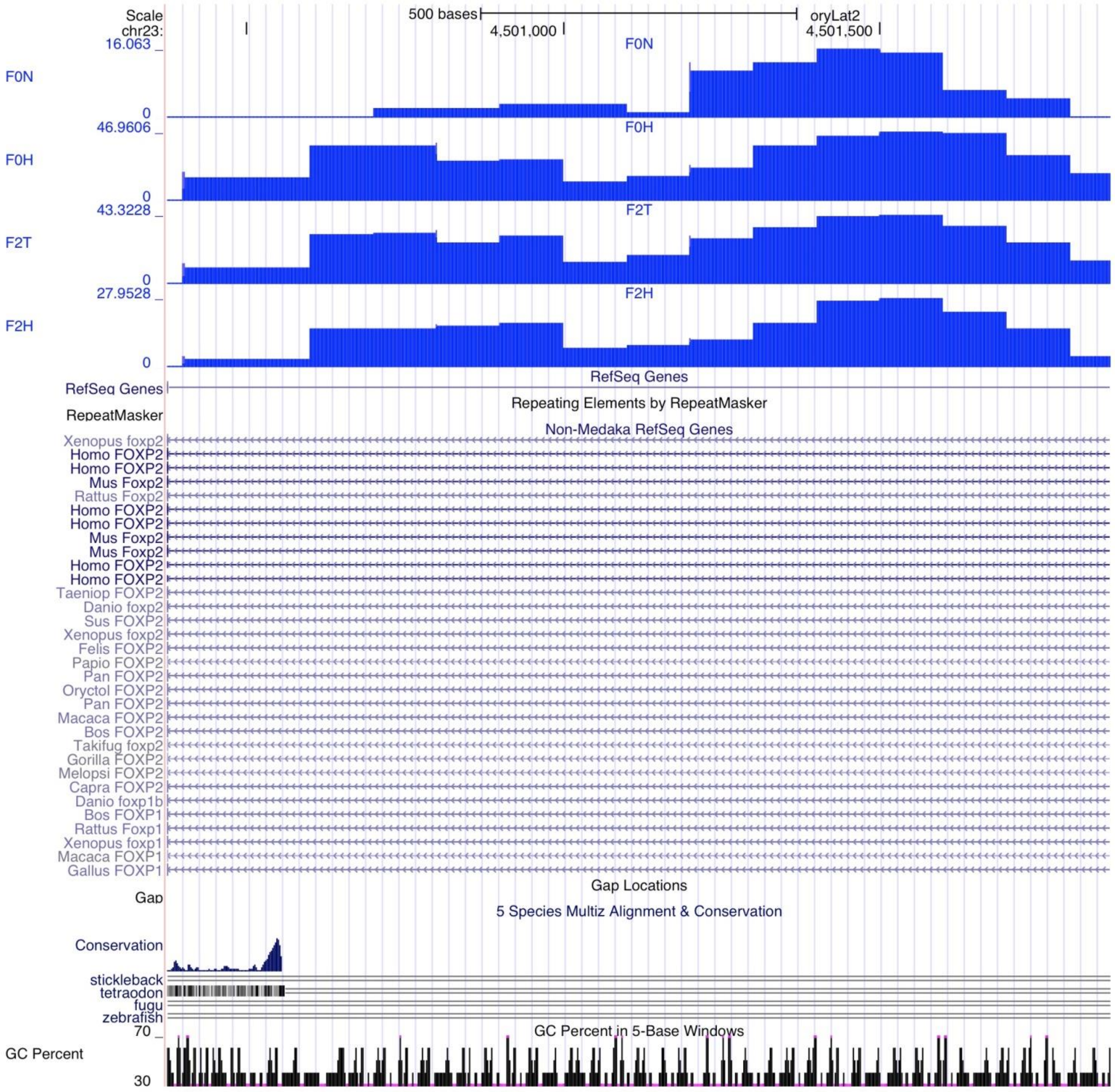


**Supplementary Fig. 7. Differentially methylated regions of FOXP2 in the sperm of generations F0 and F2.** A snapshot of MeDIP-seq results obtained using the UCSC genome browser for the genome-wide analysis. The box plot represents DNA methylation signals along the chromosome. Cut-off criteria were calculated using EdgeR. FOXP2 displayed a 1,489-bp region in the non-coding regions of F0H, F2T and F2H that was hypomethylated compared to the normoxic control groups ($n = 10$; $p < 0.01$).

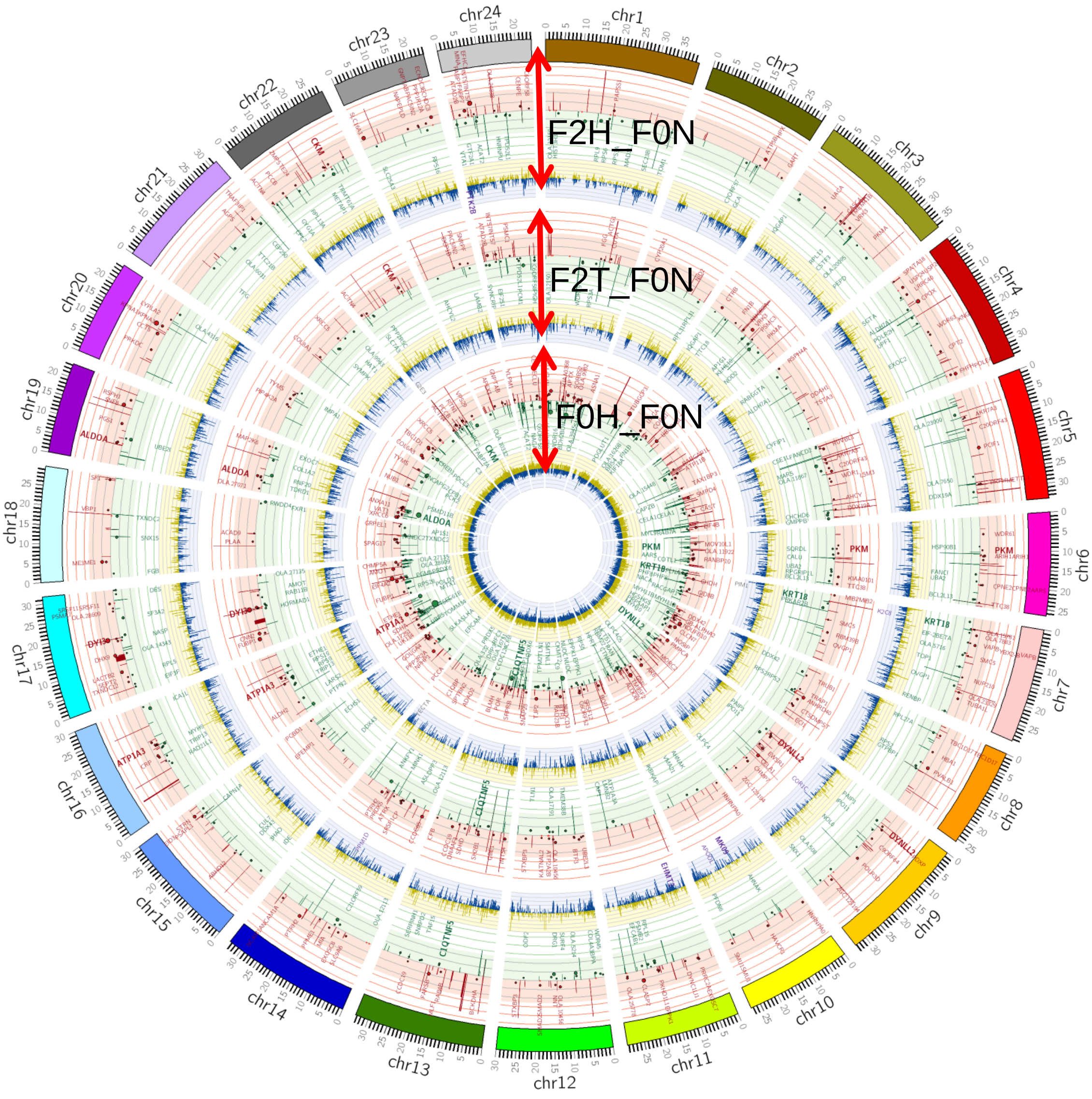


**Supplementary Fig. 8. Circos diagram describing 24 chromosomes of marine medaka.** Red and green dots represent upregulated and downregulated proteins, respectively. Red and green lines represent upregulated and downregulated genes, respectively. Yellow and blue lines represent hypermethylation and hypomethylation, respectively (n = 10).
